# Rare Endobronchial Presentation of Diffuse Large B‐Cell Lymphoma in a Rural Midwestern Patient

**DOI:** 10.1155/crom/7294425

**Published:** 2026-06-30

**Authors:** Abbey Y. Jin, Deap S. Bhandal, Rony Abou-Jawde, Mohannad Abu Omar, Mahmoud Said, Kathleen Spears

**Affiliations:** ^1^ Rural Medicine Campus, University of Missouri-Kansas City, School of Medicine, St. Joseph, Missouri, USA, umkc.edu; ^2^ Medical Oncology, Mosaic Life Care, St. Joseph, Missouri, USA; ^3^ Pulmonary and Critical Care, Mosaic Life Care, St. Joseph, Missouri, USA; ^4^ Pathology, Mosaic Life Care, St. Joseph, Missouri, USA

## Abstract

We present a case of a rural, underserved patient from the rural Midwestern United States with an extremely unusual presentation of diffuse large B‐cell lymphoma (DLBCL) in an endobronchial region. DLBCL in an endobronchial region is so rare that existing literature does not report the statistical prevalence. This case is of particular interest in the context of cancer disparities, given that oncology literature documents survival differences in lymphoma patients based on place of residence and treatment. This patient is a 68‐year‐old Caucasian male with an income level below the regional poverty line and who has cardiovascular comorbidities and a 72‐pack‐year smoking history. He currently resides alone in trailer park housing with minimal local family support. Key interventional aspects that equalize rural patient oncology outcomes include proper assessment, diagnosis, and prompt treatment of unusual or unlooked‐for presentations of disease. Prior to the diagnosis, the patient presented to the hospital complaining of a cough, shortness of breath, nausea, and generalized weakness. The chest X‐ray and CT revealed a suspicious consolidation in the left upper lobe; a follow‐up bronchoscopy revealed an endobronchial lesion in the left lingulae. A biopsy of the tumor confirmed a rare endobronchial lymphoma and an active diagnosis of DLBCL. Following standard treatment with Rituxan‐CVP, the patient is currently in remission and being treated for a lingering comorbid fungal lung infection consequent to his immunosuppressed state. His case represents a successful intervention in a rural, underserved region of an exceedingly rare lymphoma presentation.

## 1. Introduction

Diffuse large B‐cell lymphomas (DLBCLs) of endobronchial origin are an extremely rare phenomenon, whose statistical prevalence has not been calculated in the present literature [1, 2]. More specifically, lymphomas of pulmonary origin make up approximately 3.6% of all cases of pulmonary cancers [3], with primary non‐Hodgkin’s lymphoma (NHL) of pulmonary origin contributing to 0.4% of malignant lymphomas [4]. To date, only isolated case reports of primary endobronchial NHL have been documented in the current literature [2, 5, 6]. Thus, this is an exceptionally rare case with diffuse endobronchial DLBCL demonstrating ground‐glass opacities of the bilateral upper lobes and intra‐abdominal extranodal involvement.

The most common type of non‐Hodgkin lymphoma is DLBCL. The molecular subtypes of DLBCL include germinal center B‐cell‐like (GCB) DLBCL, activated B‐cell‐like (ABC) DLBCL, and primary mediastinal B‐cell lymphoma (PMBL) DLBCL. GCB, originating from germinal center B‐cells, is associated with BCL‐2 gene translocations and amplification of the REL gene, while ABC, originating from postgerminal center B‐cells, is sometimes associated with Trisomy 3 on a cell level. PMBL is thought to arise from thymic B‐cells [7].

The first signs presenting in a patient with DLBCL are painless lymphadenopathy in the axillary, neck, and groin. However, lymphadenopathy is not limited to these areas. In other cases, a patient may present with extranodal lymphoma. The most common site of origin for extranodal lymphoma is the stomach [8]. Patients who have lymphoma originating from the stomach generally have a good prognosis [9]. Patients with a poor prognosis often have elevated lactate dehydrogenase levels, multiple areas of extranodal involvement, and an age of greater than 60 years [10]. Due to the atypical presentation of this patient’s DLBCL, this unique case emphasizes the need for consistent critical evaluation and holistic patient assessments, amplified in rural settings. Hospitals primarily serving rural populations often have limited resources and capacity compared to urban counterparts. This discrepancy in resources is one of the factors contributing to the worsening of cancer disparities in patients treated at rural hospitals [11]. In lymphoma cases, rural patients are more likely to receive suboptimal treatment of their tumor [12]. Thus, this case represents a successful intervention of a challenging and extremely rare presentation in a medically underserved hospital.

## 2. Case Presentation

A 68‐year‐old male with an over 30‐year history of malignant melanoma, systolic heart failure with an ejection fraction of 20%–25%, coronary artery disease, and a previous smoking history of one pack per day for 30 years (quit 2 years prior to presentation) presented to the emergency department with complaints of right hip and back pain, significant weight loss of over 22 kg over the past 6 months, generalized weakness, and nausea. He also complained of shortness of breath and a minimal cough with clear sputum. He denied a prior history of alcohol use.

On physical exam, the patient appeared cachectic, weak, and fatigued, but no focal abnormalities were noted. The patient presented with microcytic anemia with a hemoglobin of 11.3 g/dL and an MCV of 78 fL with microcytosis.

Given his presenting symptoms, a chest X‐ray was ordered which showed large left upper lobe consolidation. A subsequent chest CT angiography showed large peripheral consolidation with air bronchograms in the left upper lobe and lingula with innumerable bilateral pulmonary nodules (Figure 1). The patient underwent a diagnostic bronchoscopy which showed an endobronchial lesion obstructing the whole bronchus at the left lingulae (Figure 2). Multiple endobronchial biopsies were performed, identifying high‐grade diffuse large B‐cell NHL (DLBCL). Following this, the patient underwent an abdomen and pelvic CT which showed multiple intra‐abdominal lymph nodes along with a pathologic right iliac bone fracture. Biopsy of his right iliac bone showed diffuse proliferation of large neoplastic lymphoid cells and necrosis, indicating widespread involvement of organ systems other than the lymph nodes. Subsequently, he was diagnosed with Stage IV DLBCL. Furthermore, per biopsy of the patient’s right iliac bone performed 6 days following initial presentation, the morphology and immunohistochemical staining pattern were consistent with a germinal center subtype of DLBCL (Table 1 and Figures 3 and 4).

**Figure 1:**
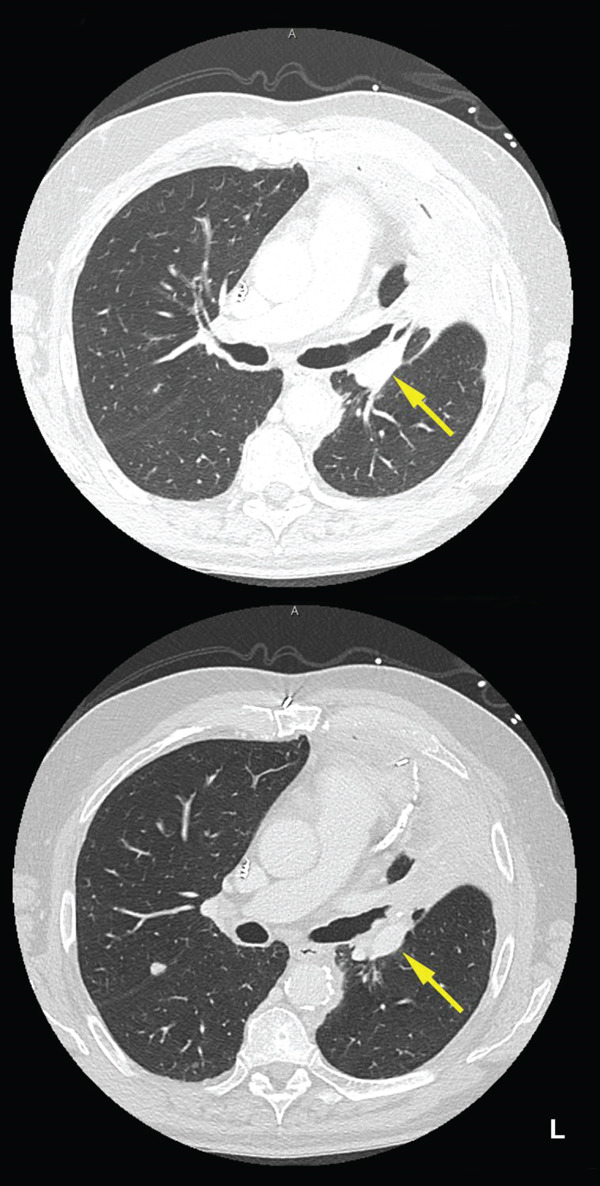
Chest CT showing large peripheral consolidation in the left upper lobe and the lingulae accompanied by air bronchogram along with atelectasis (this is concerning for an endobronchial lesion). The yellow arrows point to the peripheral consolidation accompanied by the air bronchogram.

**Figure 2:**
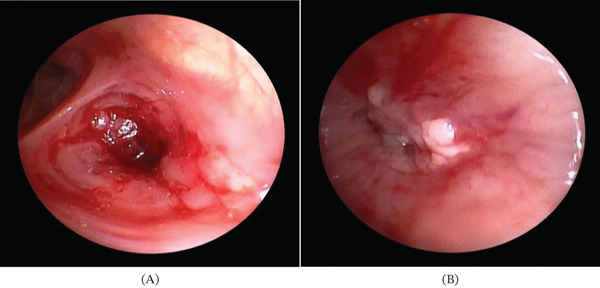
Bronchoscopy showing an endobronchial lesion (seen in B) obstructing the whole lingulae. (A) is the endobronchial lesion and shows the buildup of blood in the obstructed bronchus.

**Table 1:** Summary results from the immunohistochemical staining of the lung biopsy.

Immunological markers	Immunohistochemical staining pattern of patient	Immunohistochemical staining pattern of DLBCL
CD45	Positive	Positive
CD20	Positive	Positive
CD10	Positive	Varies (usually positive in GCB subtype, negative in ABC subtype)
BCL2	Positive	Varies
MUM1	Positive	Usually positive in ABC subtype
BCL6	Positive	Usually positive
CD3	Negative	Negative
CD5	Negative	Usually negative
BCL1	Negative	Negative (positive in mantle cell lymphoma)
CD23	Negative	Negative
Ki‐67	Near 100% proliferation fraction	High (greater than 40%)

**Figure 3:**
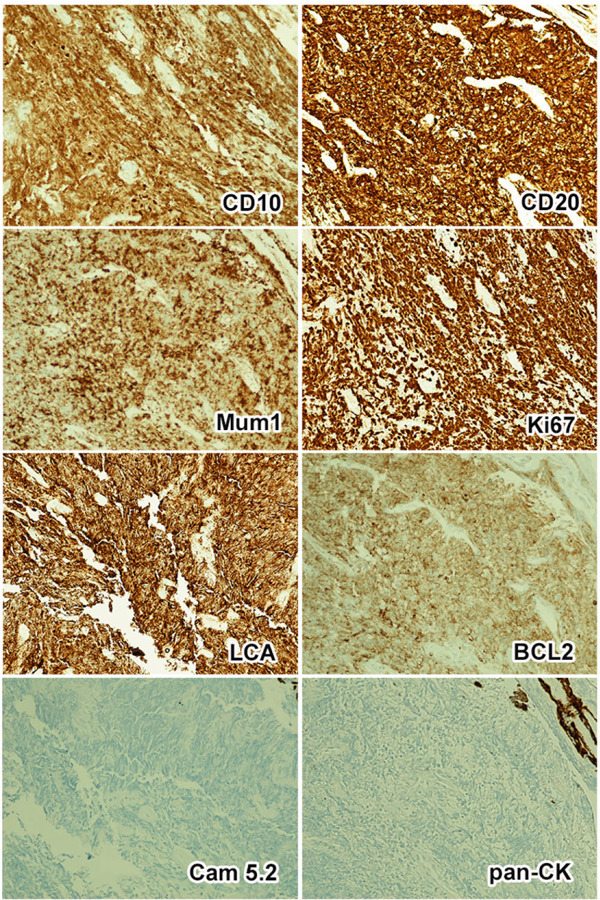
Critical immunohistochemical profile histology identifying DLCBL–germinal center subtype.

**Figure 4:**
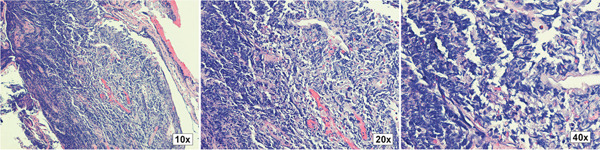
Histological profile from biopsy showing a pattern consistent with DLBCL eliminating endobronchial origin.

Interestingly, the patient’s FISH analysis revealed Trisomy 18, associated with organ abnormalities and seen in many lymphoproliferative disorders, especially BCL. There was no evidence of the patient having a double‐hit lymphoma. His high‐grade B‐cell lymphoma is consistent with DLBCL.

For treatment, the patient began his first cycle of Rituxan‐CVP (rituximab, cyclophosphamide, vincristine, and prednisone) 12 days post presentation instead of anthracycline‐based regimens because of his significantly reduced ejection fraction. He received six cycles of Rituxan‐CVP and then repeated his PET scan. His treatment focus was of palliative intent due to the aggressiveness of his cancer. His chemotherapy course was complicated by acute hypoxic respiratory failure secondary to pneumonia and perforation peritonitis at the level of the jejunum, where malignancy was found and small bowel resection was performed. Six months after his initial bronchoscopy, he had a follow‐up bronchoscopy which showed a significant regression in the endobronchial lesion.

Unfortunately, the patient did expire a few weeks after his second bronchoscopy procedure due to heart failure (Figure 5).

**Figure 5:**
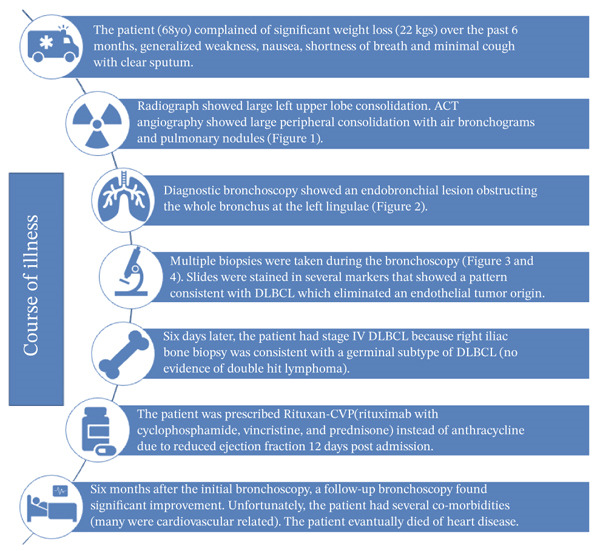
Illustration of clinical course.

## 3. Discussion

### 3.1. Pathophysiologic Mechanism of DLBCL

The novelty of this case arises from its endobronchial origin and the challenges in management in a rural setting. In fact, the original leading differential diagnosis was not lymphoma but a primary lung malignancy or melanoma per the patient’s medical history. In this case, the possibility of an extranodal manifestation of nodal DLBCL or of other anatomical sites and even pulmonary DLBCL was ruled out because of the worsening pulmonary findings with time on imaging studies, which showed a progression of pulmonary atelectasis obstruction to the development of disease outside the chest, including biopsy‐proven bone involvement, and with the finding of an endobronchial mass biopsy‐proven DLBCL. These findings solidify the fact that the lymphoma started endobronchial and then progressed.

Furthermore, the ileum or bowel was not the original source for the lymphoma because local abdominal disease was not seen on imaging studies prior to perforation.

Last, DLBCL of a primary lung origin will present with lung mass, lymphadenopathy, or, rarely, an endobronchial lesion. This case was considered to be endobronchial in origin because of the clinical presentation of worsening pulmonary atelectasis obstruction, starting with the endobronchial lymphoma progressing and then diffusely spreading within the lung and extrapulmonary sites.

However, the most common origin for DLBCL is lymph nodes rather than endobronchial [13]. The pathogenesis of DLBCL is an interplay of oncogene activation, tumor suppressor gene suppression, and involvement of other transcription factors during different stages in B‐cell differentiation. ABC is associated with a worse survival rate than GCB. CD5 expression is associated with poor prognosis, but this patient was CD5 negative [14].

### 3.2. Socioeconomic Factors

Furthermore, our patient’s management was complicated by limited resources: The patient faced this serious diagnosis while living in a trailer park alone with limited family support and subsisting on an income that was below the poverty level for his area of residence. He received care from a hospital surrounded by several rural and medically underserved areas, according to the Health Resources and Services Administration.

In lymphoma cases, rural patients are more likely to receive suboptimal treatment of their tumor [12]. While this patient did receive timely and appropriate treatment at the facility where he engaged care and was eventually able to achieve a regression of his tumor, some of these factors did delay his date of diagnosis and thus treatment initiation due to his waiting to go to the hospital until he could no longer bear his symptoms. Delay in seeing a healthcare provider is one of many reasons why patients living in rural or underserved areas tend to have more comorbidities and less positive prognoses than their urban and medically served counterparts [11, 12]. Despite limited resources and provider shortages in rural areas, our patient received optimal management.

## 4. Conclusion

While DLBCL presenting as an endobronchial lesion is rare, it can appear as an obstructive lesion in atypical locations such as the pulmonary tree and mimic lower respiratory symptom profiles. Thus, this presentation of DLBCL should be considered in the differential diagnosis of a patient with an endobronchial lesion or in patients presenting with weight loss, weakness, and difficulty breathing. Patients with multiple comorbidities need a multidisciplinary approach. Extended hospital stays, side effects from antineoplastic pharmacotherapy, and mental distress from new prognoses all impact a patient’s well‐being and mortality. Along with consulting specialties, the patient can benefit from a mutual understanding of his/her goals of care. This case represents overcoming the challenges of recognizing and treating rare lymphoma presentations in a resource‐limited setting, and its relative success underscores the importance of addressing resource distribution issues in national healthcare so healthcare providers can better serve all patient demographics.

## Funding

The study was funded by UMKC’s Rural and Tribal Research Award. This project was supported by the Health Resources and Services Administration (HRSA) of the US Department of Health and Human Services (HHS) as part of an award totaling $4 million T99HP52109 with 10% financed with nongovernmental sources.

## Disclosure

This work (Bhandal D, Jin AY, Abu‐Omar M, Said MB, Abou‐Jawde R, Spears K. “Rare Endobronchial Presentation of Diffuse Large B‐Cell Lymphoma in a Rural Midwestern Patient”) was presented as a poster at the 4th NCI Symposium on Cancer Health Disparities on April 4–5, 2023. The contents are those of the authors and do not necessarily represent the official views of, nor an endorsement, by HRSA, HHS, or the US Government.

## Conflicts of Interest

The authors declare no conflicts of interest.

## Data Availability

The data that support the findings of this study are available from the corresponding author upon reasonable request.
